# Peripheral muscle fractional tissue oxygen extraction in stable term and preterm neonates during the first 24 h after birth

**DOI:** 10.3389/fped.2023.1276769

**Published:** 2023-11-15

**Authors:** Christina H. Wolfsberger, Nina Höller, Nariae Baik-Schneditz, Bernhard Schwaberger, Ena Suppan, Lukas Mileder, Alexander Avian, Berndt Urlesberger, Gerhard Pichler

**Affiliations:** ^1^ Division of Neonatology, Department of Pediatrics and Adolescent Medicine, Medical University of Graz, Graz, Austria; ^2^ Research Unit for Neonatal Micro- and Macrocirculation, Department of Pediatrics and Adolescent Medicine, Medical University of Graz, Graz, Austria; ^3^ Institute for Medical Informatics, Statistics and Documentation, Medical University of Graz, Graz, Austria

**Keywords:** pFTOE, neonate, muscle oxygenation, reference values, near-infrared spectroscopy

## Abstract

**Background:**

Peripheral muscle fractional tissue oxygen extraction (pFTOE) represents the relative extraction of oxygen from the arterial to venous compartment, providing information about dynamic changes of oxygen delivery and oxygen consumption. The aim of the present study was to establish reference values of pFTOE during the first 24 h after birth in stable term and late preterm neonates.

**Methods:**

The present study is a *post-hoc* analysis of secondary outcome parameters of prospective observational studies. Only stable neonates without infection, asphyxia and any medical support were eligible for our analysis to obtain normal values. For measurements of peripheral muscle tissue oxygenation index (pTOI) during the first 24 h after birth in term and preterm neonates, the NIRO200/NIRO200NX was used. Arterial oxygen saturation (SpO_2_) was obtained by pulse oximetry. pFTOE was calculated out of pTOI and SpO_2_: pFTOE = (SpO_2_-pTOI)/SpO_2_. Measurements of neonates were stratified into four groups according to their respective measurement time point (6 h periods) after birth. Term and preterm neonates were analyzed separately. Mean values of measurements during the first time period (0–6 h after birth) were compared to measurements of the following time periods (second = 7–12 h, third = 13–18 h, fourth = 19–24 h after birth).

**Results:**

Two-hundred-fourty neonates (55 term and 185 late preterm neonates) had at least one peripheral muscle NIRS measurements within the first 24 h after birth. Mean gestational age and birth weight were 39.4 ± 1.1 weeks and 3360 (2860–3680)g in term neonates and 34.0 ± 1.4 weeks and 2060 (1750–2350)g in preterm neonates, respectively. In term neonates pFTOE was 0.264 (0.229–0.300), 0.228 (0.192–0.264), 0.237 (0.200–0.274) and 0.220 (0.186–0.254) in the first, second, third and fourth time period. In preterm neonates pFTOE was 0.229 (0.213–0.246), 0.225 (0.209–0.240), 0.226 (0.210–0.242) and 0.238 (0.222–0.255) in the first, second, third and fourth time period. pFTOE did not show any significant changes between the time periods, neither in term nor in preterm neonates.

**Conclusion:**

We provide reference values of pFTOE for stable term and late preterm neonates within the first 24 h after birth, which were stable when comparing four 6-h periods. These normal values are of great need for interpreting pFTOE in scientific context as well as for potential future clinical applications.

## Introduction

Peripheral muscle oxygenation, may serve as a sensitive early marker of sepsis or shock in neonates due to disturbances in microcirculation, as described in several studies within the last decades (1, 2). Peripheral muscle oxygenation can be monitored continuously and non-invasively using near-infrared spectroscopy (NIRS), which measures oxygenated hemoglobin (O_2_Hb) and deoxygenated hemoglobin (HHb) in venous (70%), capillary (20%) and arteriolar (10%) compartments (3, 4). When combining peripheral muscle NIRS monitoring with the venous occlusion method, information about oxygenation, perfusion, oxygen consumption and oxygen delivery can be obtained by different calculations based on changes in O_2_Hb and HHb during the occlusion (5, 6). One parameter obtained by this method is peripheral muscle fractional oxygen extraction (pFOE). pFOE represents the relative extraction from the arterial to venous compartment and is calculated out of peripheral muscle oxygen consumption (pVO_2_) and peripheral muscle oxygen delivery (pDO_2_) (5, 6): pFOE = pVO_2_/pDO_2_. Another parameter which can be obtained by peripheral muscle measurements is peripheral muscle fractional tissue oxygen extraction (pFTOE). pFTOE represents the relative oxygen extraction from arterial compartments to compartments of smaller arterial and venous vessels and capillaries. For calculation of this parameter, measurement of peripheral muscle tissue oxygenation index (pTOI) is necessary. pTOI is obtained by NIRS, using the spatially resolved method, which enables a non-invasive continuous measurement without venous occlusions. Using pTOI and arterial oxygen saturation (SpO_2_) pFTOE can be calculated using the following equation: pFTOE = (SpO_2_-pTOI)/SpO_2_ (7).

pFTOE has been described in several studies in preterm neonates during the first 24 h after birth (8–10). A possible comparability of pFOE and pFTOE was investigated by Hoeller et al. (11). This study described that pFOE and pFTOE show the same trend but cannot be equated, as pFOE values were found to be higher compared to pFTOE values.

As venous occlusions have some limitations including the susceptibility to movement artefacts and the limited applicability in clinical routine due to time constraints (4, 12), pFTOE is a value that might be implemented in clinical routine much easier than pFOE. However, up to now only studies on pFOE during the first 24 h after birth have been published (13). Thus, reference values of pFTOE during the first 24 h after birth are missing, which would be essential for future clinical applications of pFTOE measurements.

Therefore, the aim of the present study was to establish reference values of pFTOE during the first 24 h after birth in stable term and late preterm neonates.

## Methods

### Study design and analyses

In this *post-hoc* analysis, secondary outcome parameters obtained in five prospective observational studies with peripheral muscle oxygenation measurements during the first 24 h after birth were analyzed (10, 14, 15). These studies were conducted between January 2008 and December 2022 at the Division of Neonatology, Medical University of Graz, Austria. All studies were approved by the Regional Committee on Biomedical Research Ethics (EC numbers: 19–291 ex 07/08, 21–149 ex 09/10, 23–402 ex 10/11, 25–237 ex 12/13 and 33–161 ex 20/21). Written parental consent was obtained prior to patient inclusion in all studies.

To establish reference values for pFTOE during the first 24 h after birth, four time periods were defined according to the time-point of measurements of peripheral muscle oxygenation: 0–6 h after birth (starting after the first 15 min after birth) (“first time period”), 7–12 h after birth (“second time period”), 13–18 h after birth (“third time period”) and 19–24 h after birth (“fourth time period”). Mean pFTOE values of each time period were calculated for term and for preterm neonates. Values of the first “6 h period” were compared to the following measurement periods.

### Patients

Only stable neonates after admission to the neonatal intensive care unit (NICU) with available pFTOE values were included into our analysis to define normal values. Neonates with intensive medical (inotropes or vasopressors) or invasive and non-invasive respiratory support, perinatal asphyxia (defined as an umbilical artery pH below 7.00) or severe congenital malformations were excluded. Furthermore, neonates with clinical signs of infection, defined as C-reactive protein values higher than 10 mg/L, leucocyte counts above 34000 /µl, immature-to-total neutrophil ratio > 0.20 or positive blood cultures during the first 24 h after birth were excluded.

### Study protocols of included prospective observational studies

In one (EC number: 33–161 ex 20/21) out of the five included studies with pFTOE measurements, peripheral muscle measurements were performed within the first six hours after birth by five short reapplications. The mean pFTOE value of these reapplications was calculated for further analysis.

Peripheral muscle oxygenation measurements in the other four prospective observational studies were performed continuously for a varying time period within the first 24 h after birth. Out of these measurements, the mean pFTOE for each “6 h time period” was calculated and used for further analysis.

#### Medical history

Demographic data including sex, birth weight, gestational age, Apgar scores, umbilical artery pH and mode of delivery were documented.

#### Measurements during the first 24 h after birth

Peripheral muscle oxygenation monitoring with NIRS, as mentioned above, were conducted when the term or preterm neonate were in a stable condition at the NICU, at least 15 min after birth. Measurements of pTOI were performed either with continuous measurements during the first 24 h after birth, with a fixed NIRS probe on the right forearm, or with five reapplications, according to the study protocol. For reapplications, the NIRS probe was held gently by the examiner on the neonates’ right forearm for approximately 30 s and was then removed for a 10 s period. After that, the sensor was reapplied for further four times in the same position. The mean value of the five peripheral muscle measurements was documented.

In all prospective observational studies, peripheral muscle oxygenation measurements with NIRS were performed according to already published quality criteria and recommendations (12), using the NIRO 200 or NIRO 200NX device (Hamamatsu Photonics, Hamamatsu, Shizuoka, Japan). The interoptode distance for peripheral muscle oxygenation measurements was chosen in accordance to the birth weight of the neonate: 3.0 cm in neonates with a birth weight >1500 g and 2.0 cm in preterm neonates with a birth weight <1500 g. The thickness of the subcutaneous fat tissue and the diameter of the forearm were evaluated by ultrasound using the GE Logiq S8 (GE Health Care, Chicago, United States). Routine monitoring parameters including SpO_2_ and heart rate (HR) were continuously measured with pulse oximetry using either the IntelliVue MP50 or MX750 monitor (Philips, Eindhoven, The Netherlands). Non-invasively measured mean arterial blood pressure (MABP) was documented at least once in a stable condition at the NICU within the first 24 h after birth, using either the IntelliVue MP50 or MX750 monitor (Philips, Eindhoven, The Netherlands). Peripheral and rectal body temperature were measured using a skin and rectal probe, respectively. All monitoring data were stored in the polygraphic system “alpha-trace digital MM” (B.E.S.T Medical Systems, Vienna, Austria) for subsequent analysis.

### Peripheral muscle fractional tissue oxygen extraction (pFTOE)

pFTOE was calculated out of SpO_2_ and pTOI using the following equation (7):

$$\mathrm{pFTOE}=\frac{\left({\mathrm{SpO}}_{2}-\mathrm{pTOI}\right)}{{\mathrm{SpO}}_{2}}$$

### Statistical analysis

Demographic data were described as absolute and relative numbers, mean ± SD or median IQR, as appropriate. Courses of pFTOE, pTOI, SpO_2_ and HR within the first 24 h after birth were analysed using a linear mixed model with fixed effects for time and group (term vs preterm neonates). A first order autoregressive covariance structure was used. Post hoc analysis for group differences at each time period was performed. Results according to this analysis are presented as estimated means and 95% confidence intervals. A *p* value < 0.05 was considered statistically significant. The statistical analyses were performed using IBM SPSS Statistics 28.0.1 (IBM Corporation; Armonk, USA).

## Results

**Figure 1:**
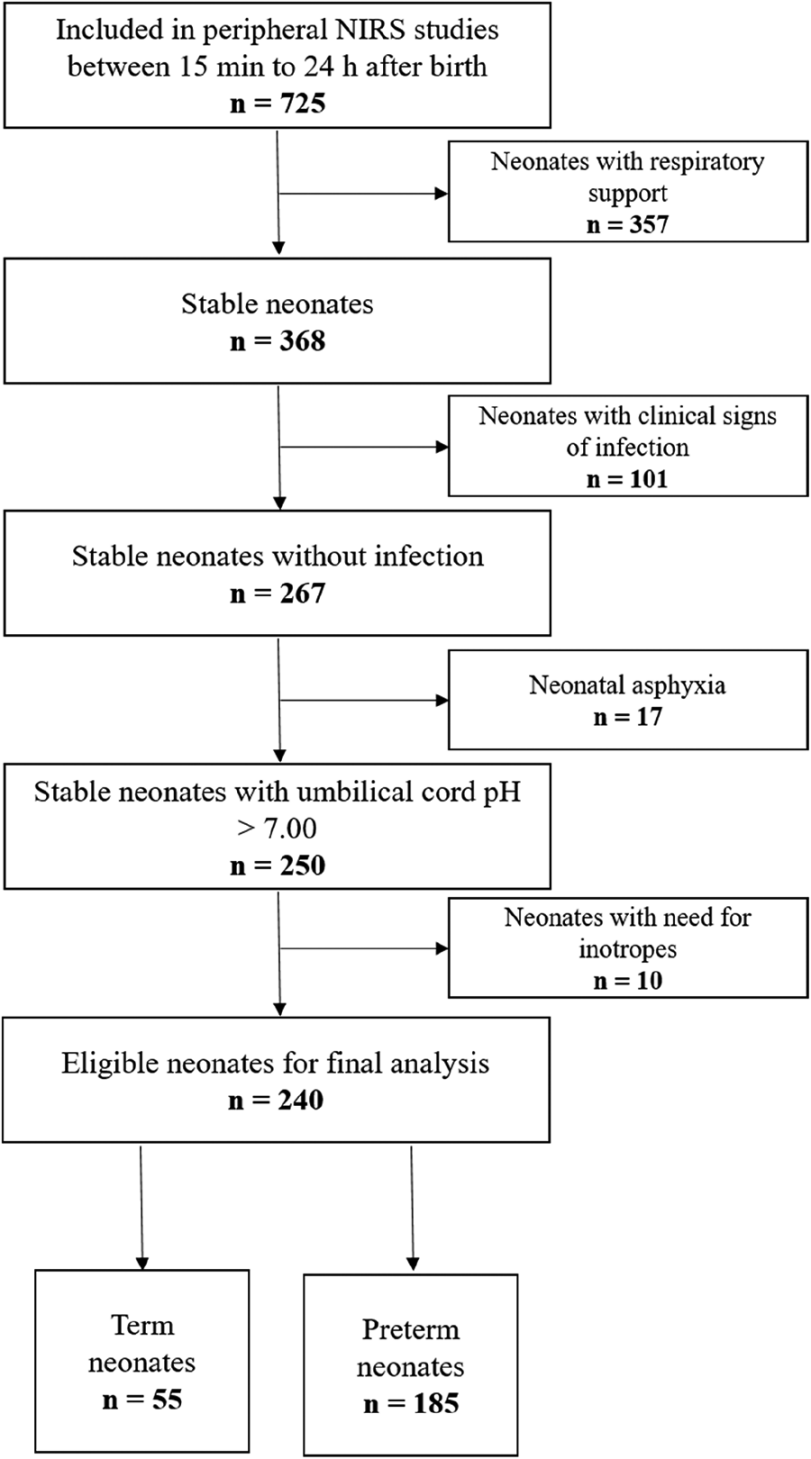
Study flow chart of term and preterm neonates with pFTOE measurements after admission to the neonatal intensive care unit during the first 24 h after birth.

Seven-hundred-twenty-five term and late preterm neonates were included in the five prospective observational studies. For the present study, we excluded 357 neonates receiving respiratory support, 101 neonates with clinical and/or laboratory signs of infection, 17 neonates with an umbilical artery pH of <7.00 and ten neonates receiving inotropes or vasopressors. Finally, 240 neonates, 55 term and 185 late preterm neonates, were eligible for the final analysis (Figure 1). Demographic data of the included term and preterm neonates are presented in Table 1. Preterm neonates were admitted to the NICU due to gestational age <34 weeks and/or a birth weight <2000 g (*n* = 58).

**Table 1:** Demographic data of term and preterm neonates with pFTOE measurements during the first 24 h after birth.

	Term neonates *n* = 55	Preterm neonates *n* = 185
Gestational age [weeks]	39.4 ± 1.1	34.0 ± 1.4
Birth weight [grams]	3360 (2860–3680)	2060 (1750–2350)
Female sex	21 (38.2%)	71 (38.4%)
Apgar 1 min	8 (8–9)	9 (8–9)
Apgar 5 min	9 (8–10)	9 (9–10)
Apgar 10 min	10 (9–10)	10 (9–10)
Umbilical artery pH	7.28 (7.20–7.31)	7.31 (7.26–7.33)
Mean arterial blood pressure [mmHg]	46.1 ± 7.9	41.1 ± 6.1
Peripheral body temperature [°C]	35.4 (34.6–36.0)	36.2 (35.7–37.0)
Rectal body temperature [°C]	37.0 (36.8–37.2)	37.0 (36.8–37.2)
Diameter right forearm [cm]	3.0 (2.8–3.5)	2.6 (2.3–2.8)
Subcutaneous fat thickness right forearm [cm]	0.34 (0.31–0.46)	0.27 (0.23–0.35)

Data are presented as *n* (%), mean ± SD or median (IQR).

**Figure 2:**
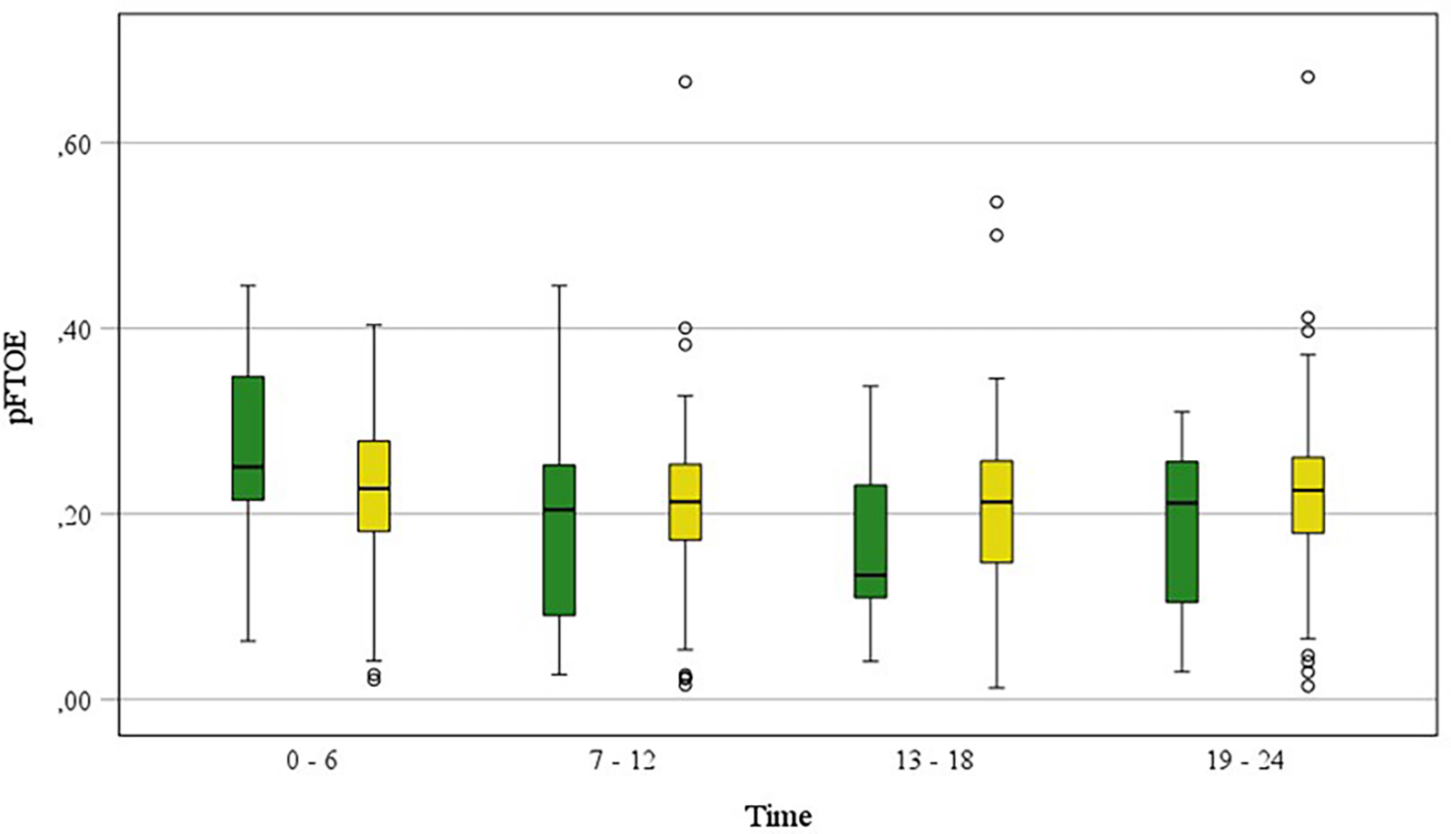
pFTOE measured with NIRS in stable term and preterm neonates during the first 24 h after birth, grouped in “6 h time periods” (“0–6 h”, “7–12 h”, “13–18 h”, “19–24 h”). Term neonates are displayed in green blots and preterm neonates in yellow blots. Values are presented as median (IQR).

**Figure 3:**
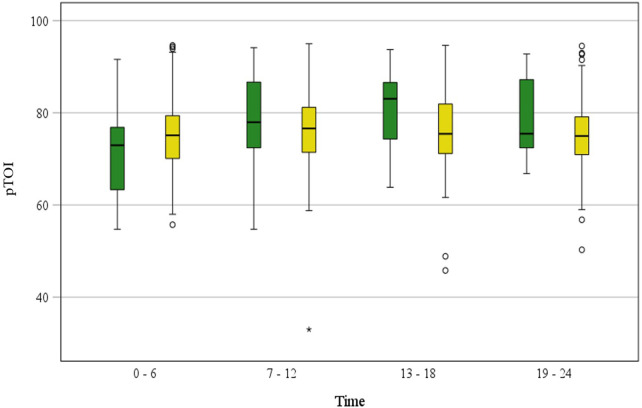
pTOI measured with NIRS in stable term and preterm neonates during the first 24 h after birth, grouped in “6 h time periods” (“0–6 h”, “7–12 h”, “13–18 h”, “19–24 h”). Term neonates are displayed in green blots and preterm neonates in yellow blots. Values are presented as median (IQR).

pFTOE and pTOI as well as routine monitoring variables are presented in Table 2. pFTOE did not differ between term and preterm during the first 24 h after birth (Table 2, Figure 2) (*p* = 0.591). Furthermore, there were no significant differences between all four time periods in term and preterm neonates (*p* = 0.303) and courses of pFTOE were similar between term and preterm neonates (*p* = 0.137). According to the model the highest value of pFTOE in term neonates was observed in the first time period [estimated mean: 0.264 (95% CI: 0.029–0.300)], whereas in preterm neonates the highest pFTOE value [0.238 (0.222–0.255)] was observed in the fourth time period. Looking on each time period, also no differences in pFTOE between term and preterm neonates could be observed: “first time period” [*p* = 0.080], “second time period” [*p* = 0.866]), “third time period” [*p* = 0.597] and “fourth time period” [*p* = 0.342]) (Table 2). pTOI also showed no differences between term and preterm neonates (*p* = 0.705), no changes over time (*p* = 0.423) and comparable courses of groups (*p* = 0.125) (Figure 3).

**Table 2:** Peripheral muscle oxygenation parameters (pFTOE and pTOI) and routine monitoring parameters of stable term and preterm neonates with peripheral muscle oxygenation measurements during the first 24 h after birth.

	First (0–6 h) time period	Second (7–12 h) time period	Third (13–18 h) time period	Fourth (19–24 h) time period
Term neonates				
Number of measurements	*n* = 20	*n* = 15	*n* = 12	*n* = 22
pFTOE	0.264 (0.229–0.300)	0.228 (0.192–0.264)	0.237 (0.200–0.274)	0.220 (0.186–0.254)
pTOI [%]	71.5 (68.2–74.8)	74.3 (70.9–77.6)	74.0 (70.5–77.5)	75.9 (72.7–79.1)
HR [bpm]	124 (119–129)	125 (120–129)	129 (124–134)	130 (125–135)
SpO_2_ [%]	97 (96–98)	97 (96–97)	97 (96–98)	97 (96–98)
Preterm neonates				
Number of measurements	*n* = 92	*n* = 86	*n* = 90	*n* = 94
pFTOE	0.229 (0.213–0.246)	0.225 (0.209–0.240)	0.226 (0.210–0.242)	0.238 (0.222–0.255)
pTOI [%]	74.6 (73.1–76.0)	74.9 (73.4–76.3)	74.6 (73.2–76.1)	73.6 (72.1–75.1)
HR [bpm]	140 (138–142)	136 (134–139)*	135 (133–137)*	136 (134–138)*
SpO_2_ [%]	97 (96–97)	97 (96–97)	96 (96–97)	96 (96–97)*

HR, heart rate; pFTOE, peripheral muscle fractional tissue oxygen extraction; pTOI, peripheral muscle tissue oxygenation index; SpO_2_, arterial oxygen saturation.

pFTOE is displayed as mean (95% CI).

* Statistically significant (*p* < 0.05) (comparison of the first “6 h time period” to the following ones).

In HR significant differences between term and preterm neonates (*p* < 0.001) with higher values in preterm neonates and differences in the course of HR (*p* = 0.010) between term and preterm neonates could be observed. While in term neonates HR did not change over time, in preterm neonates HR decreased significantly from the first to the second (*p* < 0.001), to the third (*p* < 0.001) and to the fourth time period (*p* = 0.002). Although SpO_2_ showed no significant change over time (*p* = 0.309), difference between groups (*p* = 0.138) and comparable courses of groups (*p* = 0.260), in *post hoc* analysis a statistically significant change in SpO_2_ of preterm neonates comparing the first time period to the fourth time period (*p* = 0.031) was observed (Table 2).

## Discussion

This is the first study describing reference values of pFTOE during the first 24 h after birth in stable term and late preterm neonates. Besides the identification of reference values, we found no changes in pFTOE behaviour over the four defined “6 h time periods”.

The pFTOE value of 0.264 during the first period in stable term neonates is comparable to already published data by Pichler et al. (16). Timing of the measurement, however, was different from ours (mean of 106 h after birth) (16). Furthermore, in the study by Pichler et al. (16), term and moderate-to-late preterm neonates with respiratory and medical support were also included, too. Comparing, however, pFTOE values in the fourth time period of term (0.220) and preterm neonates (0.238) of our present study with the work published by Pichler et al. (16), differences between the two studies can be observed. This observation may be based on the above mentioned differences in gestational age, need for medical/respiratory support and/or the postnatal age at measurement.

Fujioka et al. (17) presented pFTOE data during the first 72 h after birth of 0.25 ± 0.06 in term neonates and of 0.25 ± 0.06 in preterm neonates measured with the time resolved NIRS technique. Although, the measurement period, the utilized NIRS device/technique and the study cohort of neonates with and without need for medical and respiratory support, stated in the study by Fujioka et al., were different from ours, pFTOE values are still comparable. This may be explained by minimal changes of pFTOE after the initial fetal-to-neonatal transition period, with stable pFTOE values in later periods. Furthermore, it may be assumed that in neonates with appropriate medical support, no differences in oxygen supply to and/or consumption of peripheral muscle can be detected.

In contrast to our present pFTOE within the first time period, Bruckner et al. (18) published pFTOE values of median (IQR) 0.08 (0.04–0.15) in term and preterm neonates with a mean gestational age of 34 weeks within the first four to six hours after birth. These values were noticeably lower compared with pFTOE in our study [0.26 (95%CI 0.229–0.300)]. This observed discrepancy may be based on the fact, that peripheral regional oxygen saturation was clearly higher in the study by Bruckner et al. [87% (80–92)] compared to the pTOI in our study (term neonates 71.5% [68.2–74.8]; preterm neonates 74.6% [73.1–76.0]). The differences in peripheral muscle oxygen saturation and consequently in pFTOE between Bruckner et al.’s study (18) and our work may be explained by the different NIRS devices used (INVOS versus NIRO 200/NIRO 200NX), since the INVOS device shows higher tissue oxygen saturation compared to NIRO devices (19, 20). Direct comparisons of these two devices have revealed a difference of up to ten percent, especially with increasing tissue oxygen saturation (20).

Another study presenting pFTOE values was published by Ergenekon et al. (21) with median pFTOE values of 0.34 (0.19–0.47) before exchange transfusion and of 0.36 (0.23–0.48) after exchange transfusion in term neonates with polycythemia within 24 h after birth. Higher values of pFTOE were observed by Ergenekon et al. (21) compared to our results for term neonates. This discrepancy may be explained by different factors. Firstly, polycythemia influences viscosity and leading to a potential influence on oxygen extraction from the tissue. Secondly, Ergenekon et al. (21) performed measurements on the calf, whereby in our present study, measurements were performed on the forearm of the neonate. Thirdly, the interoptode distance used by Ergenekon et al. (21) was 4.0 cm, which is also higher than in our present study with an interoptode distance of 3.0 cm in neonates with a birth weight >1500 g. Fourthly, only 15 neonates were included and analyzed by Ergenekon et al. (21). As the number of included neonates is 16 times higher in our study and quality criteria to increase reproducibility, published by Pichler et al. (12), were taken into account for all measurements, we assume that our data are more representative as normal values.

pFTOE did not show any significant changes when comparing the first “6 h time period” to the following time periods within the first 24 h after birth in our study. In preterm neonates pFTOE remained almost stable during the first three time periods, with a slight increase in pFTOE afterwards. Similarly, Wolfsberger et al. (13) observed changes of pFOE in stable preterm neonates during the first 24 h after birth, comparing the first “6 h time period” to the following ones. They described a decrease of pFOE from the first to the third time period and afterwards a significant increase comparing the first to the fourth time period. However, when comparing pFTOE of our present study [0.229 (0.213–0.246)] during the first “6 h time period” to pFOE (13) [0.35 (0.29–0.40)] at the same time period, higher values were observed for pFOE. Differences between and comparability of pFTOE and pFOE have already been investigated by Hoeller et al. (11), who described the same trend of lower pFTOE values compared to pFOE values within the first few days after birth. This emphasizes that, even though both parameters describe the estimation of oxygen extraction in the tissue, pFTOE and pFOE cannot be used interchangeably. While pFOE reflects the oxygen extraction from the arterial to venous compartment, pFTOE reflects a mixed saturation compartment (venules, arterioles and capillaries).

Possible relevance for pFTOE in clinical practice and therefore the need for reference values have been described in associations between pFTOE and cardiac function (18), partial exchange transfusion (21), ventilator modes (22), and carbon dioxide (CO_2_) (23).

The potential influence of respiratory support on pFTOE during initial fetal-to-neonatal transition in neonates has already been described (24). After initial fetal-to-neonatal transition period, Ericksen et al. (22) investigated the influence of changes in mode of invasive and non-invasive ventilation at a median of 14.6 days after birth in preterm neonates, without any statistically significance of these changes on pFTOE. Unfortunately, pFTOE values were not provided in the same form as they are in our present study. Nevertheless, pFTOE values displayed in the figure by Ericksen et al. (22), can be compared to our values presented in this work. Besides respiratory support, hematocrit can also influence pFTOE. Therefore, changes of pFTOE in neonates with polycythemia and need for partial exchange transfusion were investigated before and after transfusion (21), showing an increase in cerebral oxygenation and a decrease in pFTOE during the first 24 h after birth in 15 term neonates.

The potential effect of changes in CO_2_ levels, measured by a transcutaneous probe, on pFTOE has been observed in 13 neonates with a birth weight below 1500 g (23). Twenty-three measurements were performed during the first three days after birth and a significant negative correlation of CO_2_ and pFTOE, independent of MABP, was observed. In this study, 23 individual pFTOE values were described from each single measurement, including five measurements during the first 24 h after birth. These pFTOE values proved to be comparable to our results of preterm neonates.

### Strengths and limitations

The strengths of the study are that we focused on a homogeneous population, i.e., stable neonates without respiratory or cardio-circulatory support, and that the sample size, especially of preterm neonates, was large. The first limitation is that in term neonates the sample size was lower, however being still representative in comparison to previous studies. The second limitation is that less than 40% of the included infants were female, whereby this is below the rate of admitted female neonates (∼48%) to the NICU (25). The third limitation is that pFTOE was not calculated in all of the included term and preterm neonates in each “6 h period” during the first 24 h after birth. Therefore, changes in pFTOE over time should be interpreted with caution.

## Conclusion

Reference values of pFTOE during the first 24 h after birth for four “6 h time periods” were defined in this study. Furthermore, pFTOE measured during the first 24 h after birth, showed no statistically significant changes over time during the first 24 h after birth. These normal values of pFTOE are of great need for interpreting pFTOE in scientific context as well as for potential future clinical applications.

## Data Availability

The dataset generated and analysed during the current study are not publicly available due to their containing information that could compromise the privacy of research participants, but are available from the corresponding author on reasonable request.
